# Inter- and intraspecific diversity of food legumes among households and communities in Ethiopia

**DOI:** 10.1371/journal.pone.0227074

**Published:** 2019-12-23

**Authors:** Morgan L. Ruelle, Zemede Asfaw, Asmare Dejen, Sarah Tewolde-Berhan, Amsalu Nebiyu, Tamado Tana, Alison G. Power

**Affiliations:** 1 Department of Ecology & Evolutionary Biology, Cornell University, Ithaca, New York, United States of America; 2 International Development, Community & Environment Department, Clark University, Worcester, Massachusetts, United States of America; 3 Department of Plant Biology and Biodiversity Management, Addis Ababa University, Addis Ababa, Ethiopia; 4 Department of Agricultural Entomology, Wollo University, Dessie, Ethiopia; 5 Department of Food Science and Post-Harvest Technology, Mekelle University, Mekelle, Ethiopia; 6 Department of Horticulture and Plant Sciences, Jimma University, Jimma, Ethiopia; 7 School of Plant Sciences, Haramaya University, Haramaya, Ethiopia; Università Politecnica delle Marche, ITALY

## Abstract

Smallholders throughout sub-Saharan Africa produce legume crops as sources of food, fodder, and cash income, as well as to improve soil fertility. Ethiopian farmers have developed diverse legume varieties that enable adaptation to changing agroecological and sociocultural conditions. However, over the past several decades, as farm sizes declined and extension services promoted new varieties developed by plant breeders, changes in legume diversity have not been monitored. Based on interviews with smallholder farmers (n = 1296), we investigated the status of inter- and intraspecific legume diversity in major production areas of Ethiopia for five food legumes: common bean (*Phaseolus vulgaris* L.), field pea (*Pisum sativum* L.), faba bean (*Vicia faba* L.), groundnut (*Arachis hypogaea* L.) and fenugreek (*Trigonella foenum-graecum* L.). Legume species richness increased with altitude, relative household wealth, and land area planted to legumes. The highest numbers of varieties were found for common bean, followed by field pea, faba bean, groundnut and fenugreek. The average number of varieties planted per household was low (ranging from 1 to 2) and often much lower than the number reported in the same community or zone, which ranged from 2 to 18. For three out of the five species, the number of varieties significantly increased with total land area planted to legumes. Most varieties were rare, planted by less than 1/3 of farmers; however, informants accurately named varieties planted by others in the same community, demonstrating awareness of legume diversity at the community level. Given that the ability to plant multiple legume varieties is limited by land size, policies need to strengthen community-level conservation based on the diverse interests and needs of individual households.

## Introduction

In the context of food insecurity, economic instability, and climate variability, agrobiodiversity enhances the adaptive capacity and resilience of farming communities by providing alternative options under increasingly unpredictable and rapidly changing conditions [1,2]. Agrobiodiversity encompasses domesticated and non-domesticated plants, animals, and microorganisms found in agricultural landscapes, as well as the knowledge and practices developed by farmers through relations with their agroecosystem over generations [3,4]. In much of the world, agrobiodiversity is a primary source of food security based on its direct use within food systems and other contributions to agroecosystems [5,6]. However, the reduction of farm sizes and the demand for uniform and high-yielding agricultural products leads to high input monocropping systems that undermine the adaptability of farming households [7,8].

Grain legumes (species in Fabaceae grown primarily for edible seeds) are an important component of agrobiodiversity because they provide food, fodder, and cash income, as well as improved soil fertility, and thereby contribute to the livelihood security of smallholder farmers throughout the tropics [9–11]. Legume grains are rich in proteins, carbohydrates, fats, dietary fibers, vitamins and minerals [12,13]. As an alternative to animal sources of protein, a diet rich in legumes reduces the risk of many chronic diseases [11]. In mixed crop and livestock production systems, legume residues (pods, stems and leaves) are a valuable source of proteins, vitamins, and fiber for domesticated animals [14]. Furthermore, due to their mutualism with rhizobial bacteria that fix atmospheric nitrogen, legumes enhance soil fertility without the expense and negative impacts of chemical fertilizers on the environment [15]. Large and small-scale producers throughout the tropics use legumes in rotation or intercropping systems to enhance the productivity of other food and cash crops [16]. Legumes are therefore an essential component of agroecological intensification, which uses ecological principles to enhance agricultural productivity while maintaining and increasing agroecosystem services [17,18].

Ethiopia is a well-known center of botanical diversity, with nearly 5800 plant species and high levels of endemism [19]. Archaeological evidence indicates that Ethiopian agriculture dates back to the first millennium BCE [20,21], but linguistic analyses suggest a much longer tradition of up to 7,000 years [22]. The country’s rugged topography, wide range of agroecological conditions, and myriad cultural groups with distinct farming practices have led to diversification of domesticated plant and animal species [23]. Nikolai Vavilov [24] identified Ethiopia as the center of origin for many species, including one legume (cowpea, *Vigna unguiculata* (L.) Walp.) and as a secondary center of diversity for species originating in West Asia, including chickpea (*Cicer arietinum* L.), lentil (*Lens culinaris* Medik.), grasspea (*Lathyrus sativus* L.), field pea (*Pisum sativum* L.), faba bean (*Vicia faba* L.) and fenugreek (*Trigonella foenum-graecum* L.). In addition, the same agroecological and sociocultural factors have led to the diversification of legumes originally from the Americas, including common bean (*Phaseolus vulgaris* L.) and groundnut (*Arachis hypogaea* L.) [25].

Legumes are an essential component of smallholder farming systems across Ethiopia. Surveys indicate that legume production increased between the 1990s and 2010s, and export values of nine legume species rose from $25 million in 1995 to nearly $200 million in 2012 [26]. Ethiopia is currently the world’s second largest producer of faba bean [27]. Following faba bean, the highest production is observed for common bean, field pea, chickpea, grasspea, and lentil [26]. Ethiopia’s agricultural extension system, working with plant breeders at national and regional research centers, universities and NGOs, has focused on introducing new cultivars. While these cultivars can boost crop productivity under optimal conditions, it is less clear if they satisfy the diverse needs of rural farming communities, and they may not perform as expected under the marginal conditions faced by many smallholders [28,29]. Farmers’ traditional varieties, or landraces, have been selected for specific agroecological niches and in some cases have been shown to better resist insect pests, diseases and climate shocks, as well as meet the nutritional and cultural needs of Ethiopia’s diverse farming communities [30–33].

Despite the significance of Ethiopia’s legume diversity, it remains largely unstudied and unmonitored. As of 2012, the Ethiopian Biodiversity Institute (EBI, formerly the Institute of Biodiversity Conservation) reported holding 8,424 accessions of grain legumes at its national gene bank in Addis Ababa [34]. However, documentation associated with these collections rarely includes information from the farmers who grew them, so it is difficult to estimate the number of distinct varieties among them, let alone identify their advantageous traits and values. Westphal [25] conducted morphological characterization of traditional varieties collected from markets throughout the country, but did not document farmers’ knowledge about them. More recently, several ethnobotanical studies have documented the continued use of traditional legume varieties by farmers (e.g. [35,36]), but have not investigated their current status in a systematic way. To date, there has been no nationwide survey to document farmers’ knowledge and use of grain legume varieties.

As smallholders gain access to global markets and the government’s extension services disseminate new cultivars, the future of farmers’ traditional legumes is uncertain. While national production of legumes has increased [26], farmers in some parts of the country report that their use of legumes is declining due to changes in the production of cereals, which are increasingly grown using inorganic fertilizers rather than crop rotation to restore soil fertility [37]. In addition, the spread of new pests that attack legumes (e.g., *Orobanche* spp.) leads farmers to abandon their production [38]. Understanding and supporting *in situ* conservation by farmers is necessary to ensure that rural communities have access to a diverse array of legume species and varieties that can serve their needs under rapidly changing climatic and economic conditions [28,29]. Comprehensive analyses that incorporate farmers’ knowledge are needed to understand the values of legume diversity to farmers, establish a baseline from which to monitor changes in their abundance and map their distribution among households and communities. In addition, there is a need to expand local awareness and identify opportunities to build on existing expertise to enhance conservation efforts like those undertaken by the EBI and other institutions [39].

Based on interviews with smallholder farmers, we report on the inter- and intraspecific diversity (i.e., between and within species) of grain legumes across a range of agroecological and sociocultural contexts in four regions of Ethiopia. The objectives of this study were 1) to assess the impacts of multiple socioeconomic and agroecological factors on legume species diversity, 2) to measure the varietal diversity of five legume species (common bean, faba bean, field pea, fenugreek, and groundnut) in areas of major production, and 3) to evaluate the conservation of legume varieties by households and communities. Through an analysis of the status and significance of legumes in Ethiopia, we provide data to initiate long-term monitoring and a foundation for policies aimed at maintaining agrobiodiversity as a source of stability, sustainability, and resilience.

## Methods

### Ethics

The research protocol (#1605006357) was determined exempt from review by the Institutional Review Board of Cornell University. Free and informed oral consent was obtained from all participants prior to each interview.

### Study design

**Fig 1:**
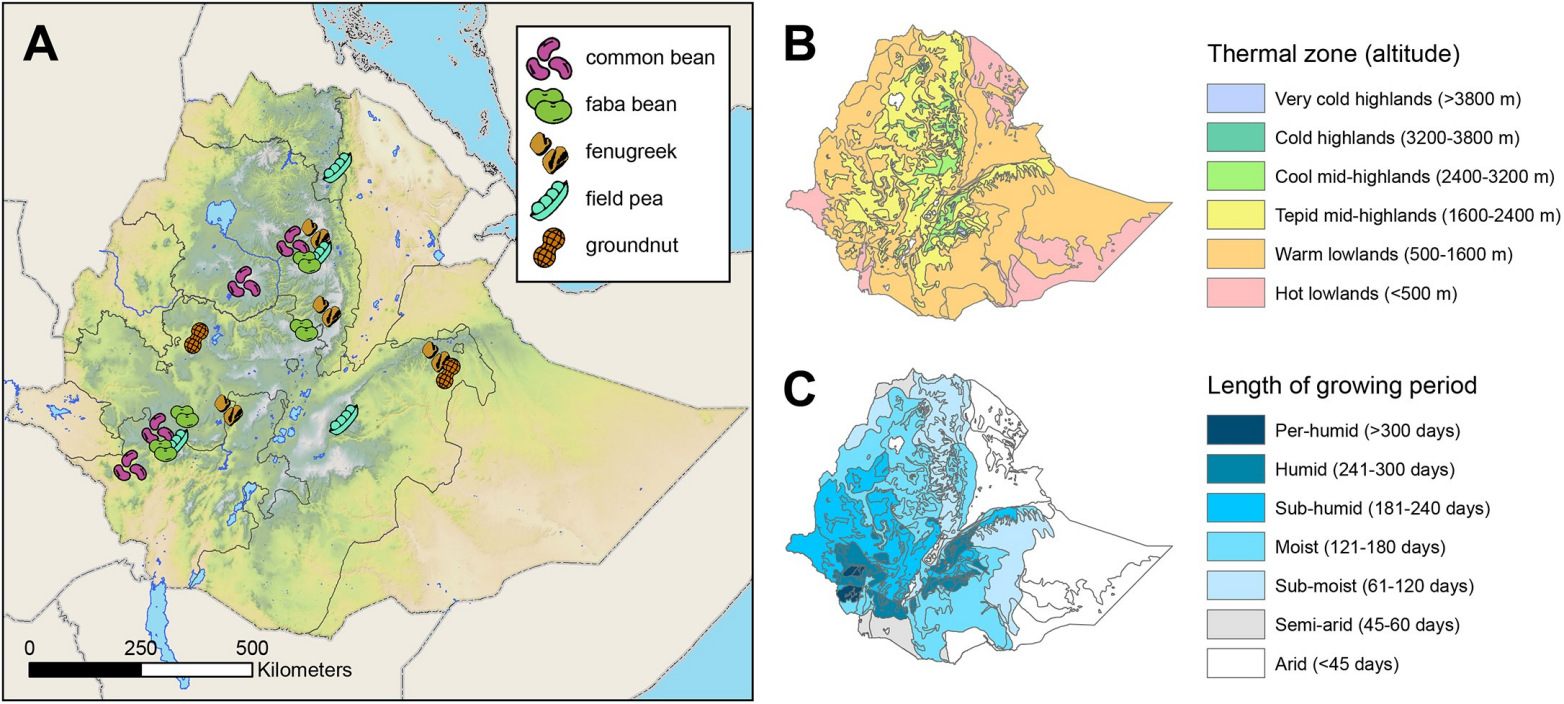
Map of study areas within Ethiopia. A) locations of surveys; each icon represents 72 structured interviews with general informants and 12 semi-structured interviews with key informants; B) thermal zones as determined by altitude; C) length of growing period (Data sources: Ethiopian Institute of Agricultural Research).

**Fig 2:**
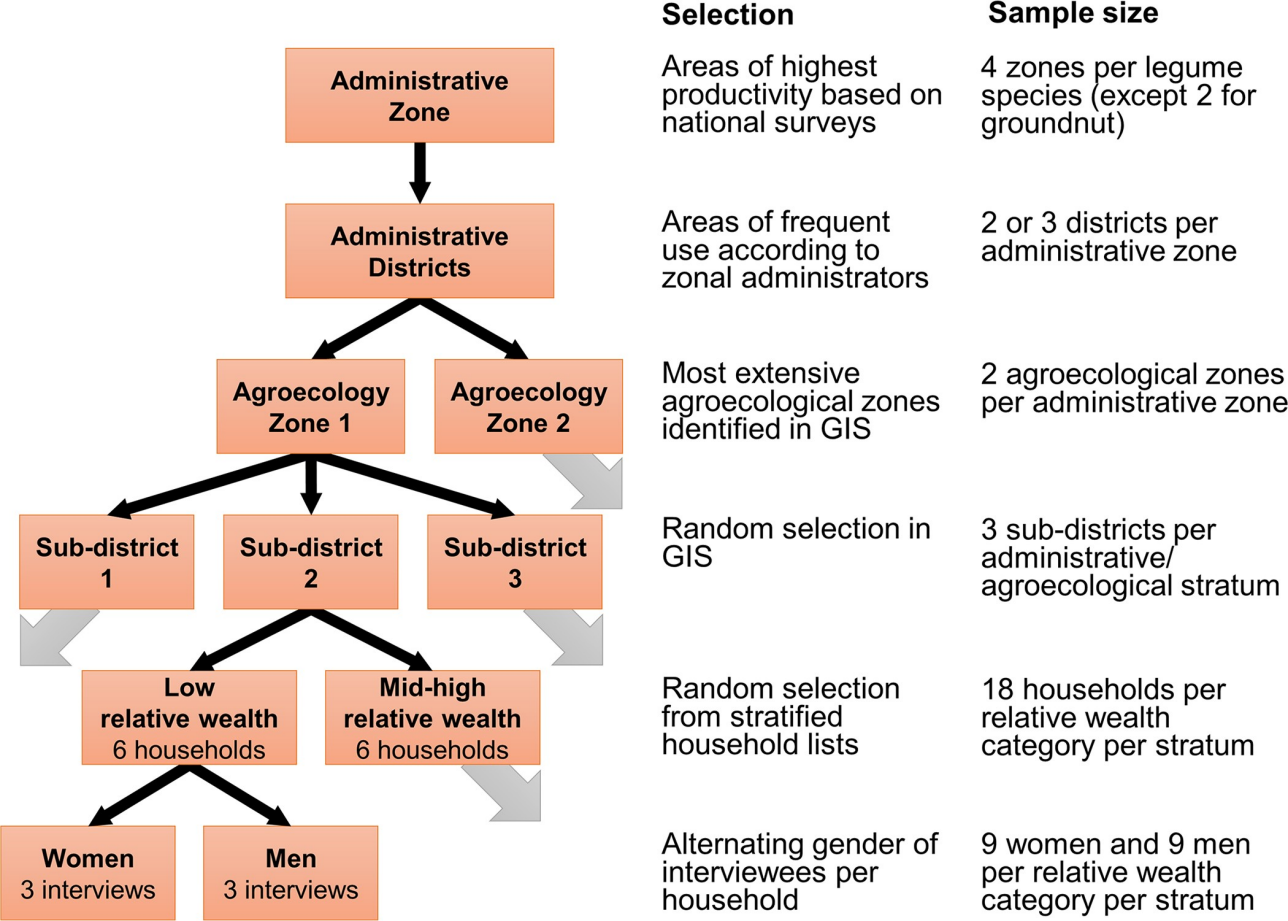
Study design. Diagram of multistage sampling process to select farming households for interviews.

Areas of high production for the target legume crops were identified based on production data from 2015/2016 [40]. Four administrative zones (hereafter called “zones”) were selected for each legume species, except for groundnut, which was surveyed in two zones that fulfilled our site selection criteria. Within each zone, local administrators and other experts were consulted to select two or three districts in which a large proportion of farmers were known to produce the target legume. Using GIS (ArcMap version 10.1, ESRI), the sub-districts (hereafter called “communities”) within each of these districts were stratified by agroecological conditions, following a classification system developed by Ethiopia’s Ministry of Agriculture and Rural Development [41] based on thermal zones (defined by altitude) and length of the growing season (Fig 1). Three communities were randomly selected for each combination of zone and agroecology; altogether 107 communities within 11 zones and 10 agroecologies were included in the study (S1 Table). Local officials within each community developed a list of farming households known to produce the target legume. Each list was stratified according to relative wealth (i.e., low and mid-to-high income households), and six households were randomly selected from each wealth category. Researchers selected one individual per household to serve as a general informant, including an equal number of males and females within each wealth category (Fig 2).

### Data collection

Structured interviews were used to collect information on farmers' use, management, and valuation of legume varieties (S1 Appendix). Interviews were conducted from September 2016 to January 2017 using Open Data Kit (https://opendatakit.org), a digital data collection application, on GPS-enabled mobile phones and uploaded to a secure data repository (KoBoToolbox, https://kobotoolbox.org). Spatial coordinates and altitude were recorded using the GPS on the mobile phone. Interviews included questions about all legume crop species produced by the household, followed by more in-depth questions about the target legume (S1 Data), including the names of varieties planted in the household, those planted by others in their community, and those that informants remember but are no longer planted (S2 Data).

We analyzed legume varieties according to local names. Use of the same names by farmers within the same administrative zone was assumed to denote the same variety. Researchers verified the local names of varieties during semi-structured interviews conducted with two knowledgeable key informants (one male and one female) within each community and collected a sample of seeds associated with each; these samples were deposited at the Ethiopian Biodiversity Institute (EBI) or other suitable research institution. Informants were asked if they considered each variety to be ‘traditional’ or ‘new’. In discussions with farmers, ‘traditional’ varieties were defined as having been planted by previous generations in the same household or community, whereas ‘new’ varieties included those received from extension agents or via exchange with farmers in other zones or regions. Given the possibility that some varieties that were originally received from the formal breeding system are now considered ‘traditional’ by farmers who have used them for several decades and the likelihood that some ‘new’ varieties were recently obtained from other zones where they have been planted for centuries, we cannot assume that all ‘traditional’ varieties are landraces (sensu [42]) nor that all ‘new’ varieties were improved by plant breeders. Therefore, our analyses are based on farmers’ classification of their own germplasm, and genetic research would be necessary to determine if these classifications align with scientific definitions of landraces and improved cultivars.

### Data analysis

We analyzed the distribution of legume crops across administrative zones and agroecologies based on the percent of farmers planting each species in the 2015/2016 growing season. All analyses were conducted in R (version 3.4.3) and R-Studio (version 1.0.143).

Species response curves for each legume species as a function of altitude were generated using a Generalized Additive Model (GAM) with presence/absence per farm (as reported by farmers for the 2015/16 growing season) as a binary response. To minimize bias in these models, areas where the legume had been targeted were excluded.

Legume species richness per household was based on the number of legume species planted in 2015/2016. Fenugreek was excluded from our measure of species richness because many farmers consider it a spice rather than a legume; therefore, it was not consistently mentioned by those who plant it. To identify factors associated with species richness at the household level, we developed a mixed model with random intercepts. Farmers who reported planting more than 2 ha of legumes were excluded as they are not considered smallholder farmers [43]. The target legume species, zone, and community were included as nested random effects; altitude, length of the growing season, relative wealth, gender of household head, and total area planted to legumes were included as fixed effects. Three generalized linear mixed models (GLMMs) using normal, Poisson, and zero-truncated Poisson distributions were generated and compared using the ‘lme4’ and ‘glmmTMB’ packages [44,45].

Varietal richness per zone, community, and household were based on the combined count of traditional and new varieties planted in 2015/2016. For each legume species, we developed a separate GLMM to measure the influence of agroecological and socioeconomic factors on varietal richness. Because the number of varieties per household rarely exceeded two, varietal richness was transformed to binary data, such that any household planting only one variety in 2015/2016 was coded as zero and those planting more than one variety were coded as one. Models for each species were fit to a binomial distribution using the restricted maximum likelihood (REML) approach in the ‘glmmTMB’ package. Zone and community were included in all models as nested random effects; altitude, average annual rainfall, length of the growing season, relative wealth, gender of the household head, and total area planted to legumes were used as fixed effects. Each model was optimized by step-wise elimination of fixed effects to minimize the AIC. Altitude, average annual rainfall, length of the rainy season, and relative wealth were eliminated from all models. Depending on the species, area planted to legumes and gender of the household head remained as factors influencing varietal richness. The effects of zone and community were determined by calculating the intraclass correlation coefficient using the ‘insight’ package in R [46].

We assessed the conservation status of legume varieties within each zone by calculating the fraction of farmers who reported planting it in the previous three years (2014 to 2016). Varieties planted by more than 2/3 of farmers in the zone were considered ‘high use’, those used by more than 1/3 to 2/3 of farmers ‘medium use’, and those used by 1/3 or less of ‘low use’.

Farmers were asked to name any varieties that were being planted by others within their community but not themselves. These names were compared to lists generated by others within the same community and zone to measure farmers’ knowledge of the varietal diversity available within their community.

Finally, farmers were asked to name varieties that they remembered from the past but are no longer planted in their own community. Again, we compared these names with those of varieties planted by other informants to see if any could be located within the same community or zone. Names of varieties that were said to have disappeared and were not located elsewhere were compiled and reported to the EBI.

## Results

### Interspecific diversity

**Fig 3:**
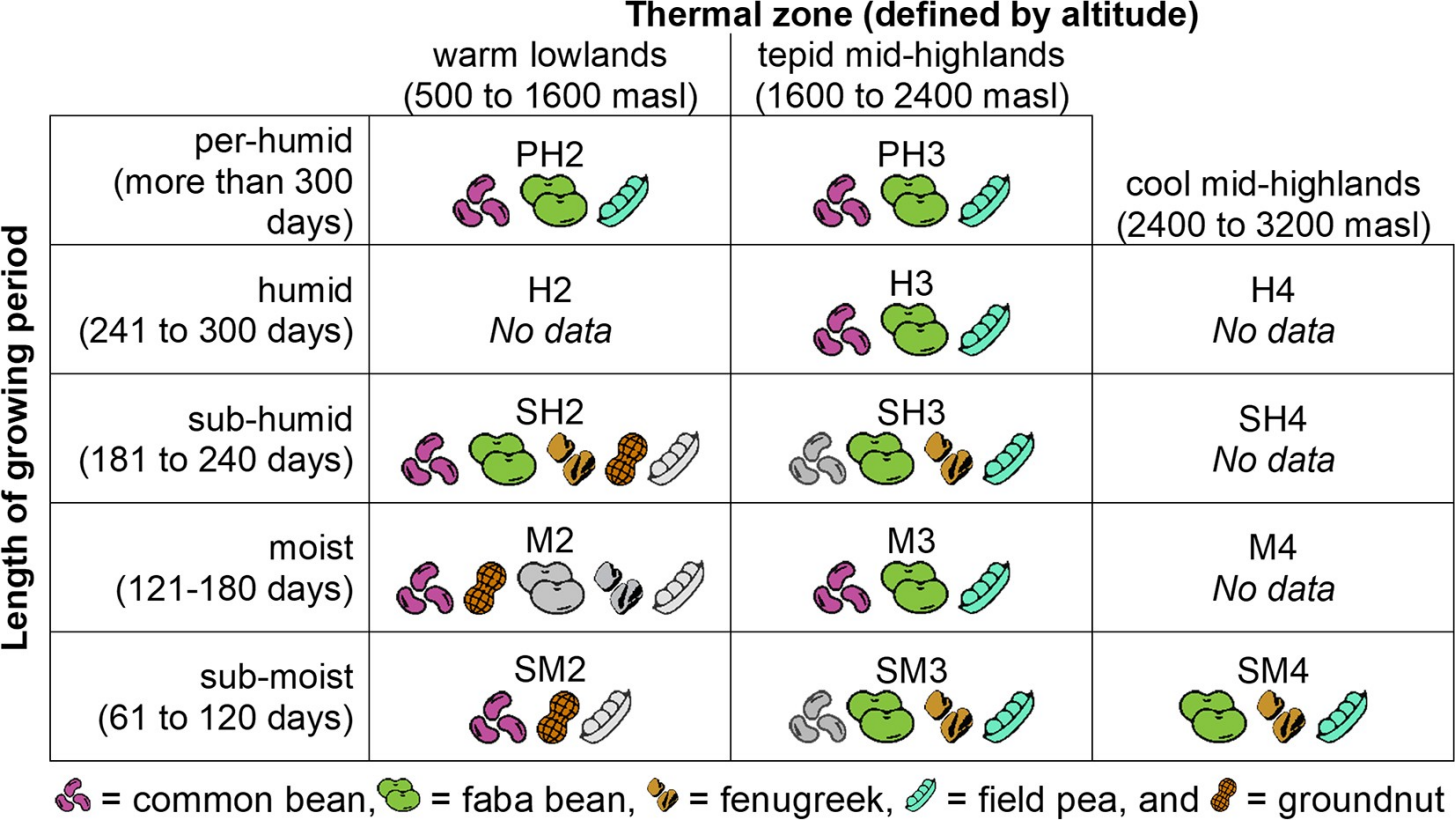
Occurrence of five legume species in different agroecologies. Each agroecology is associated with a unique alphanumeric code. Grey icons indicate that the crop was planted by fewer than 20% of interviewees.

Altogether, 12 legume species were encountered during surveys, including the five legume crops targeted, as well as chickpea, cowpea, grasspea, lentil, lima bean (*Phaseolus lunatus* L.), mung bean (*Vigna radiata* (L.) R.Wilczek) and soybean (*Glycine max* (L.) Merr.). Of the five targeted species, field pea was the most widely distributed and found in all agroecologies included (Fig 3). Faba bean was found in all but the lowest and driest agroecology (warm, sub-moist lowlands), and common bean was documented in all but the highest altitudes (cool, sub-moist mid-highlands). By contrast, groundnut was the most limited in its range, as it was observed only in the three lowland zones (sub-moist, moist, and sub-humid) where it was targeted.

**Fig 4:**
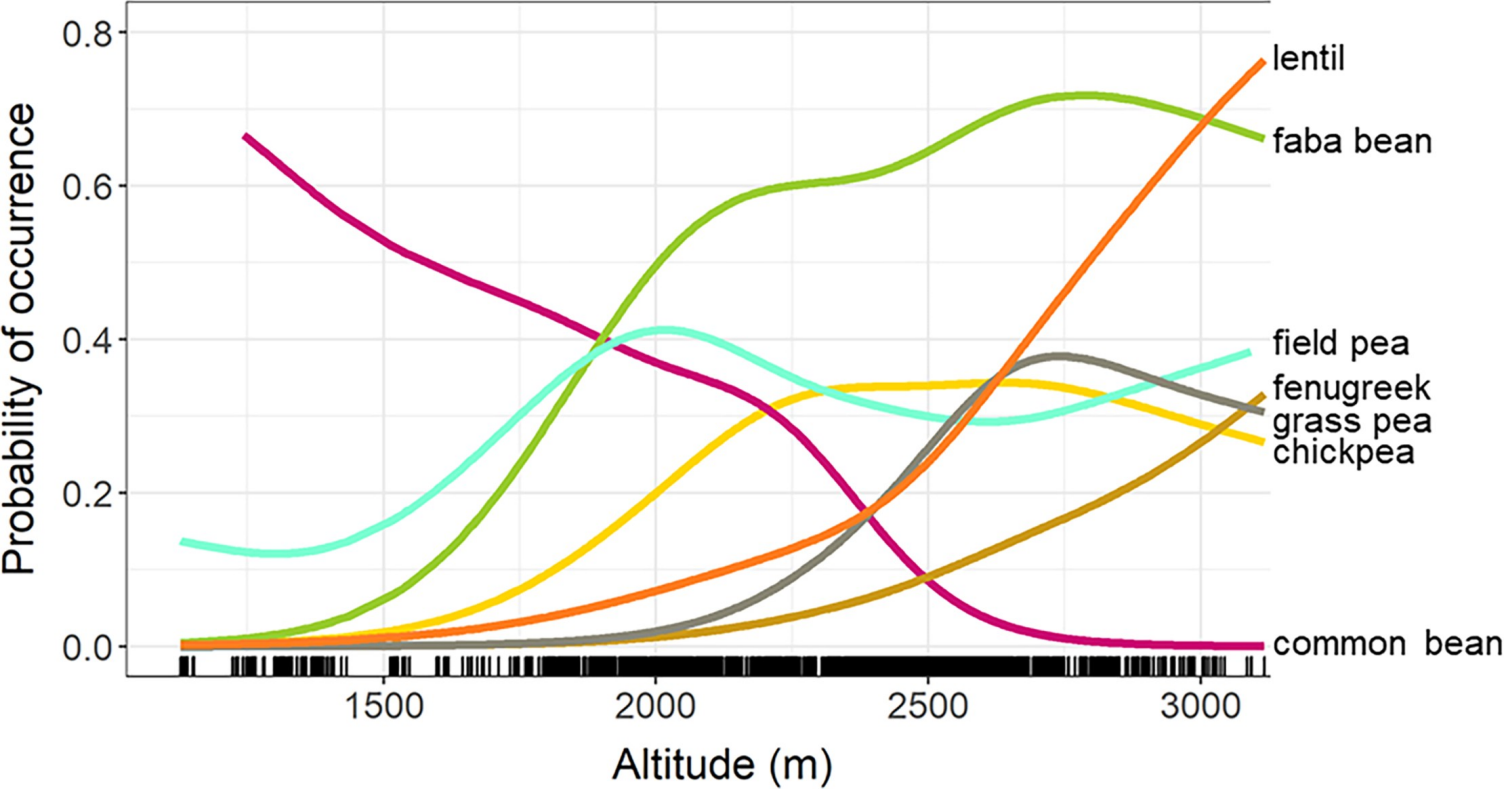
Species response curves for legume species as a function of altitude. Lines represent general additive models (GAMs) based on presence on farms during the 2015/16 growing season.

Species response curves generated from presence-absence data indicate that more legume species were found at mid to high altitudes (Fig 4). Groundnut was excluded from this analysis because it was only observed where it was targeted, but it is expected to be more common at low than high altitudes. Otherwise, common bean was the only species that was more frequently observed at low than at high altitudes. Field pea and chickpea occurred most frequently at mid-altitudes (approximately 2000 to 2500 m), whereas faba bean, grasspea, lentil, and fenugreek were most likely to be found at higher altitudes.

Overall, households reported planting between one and six legume species in 2015/2016. Species richness varied according to zone and community, as indicated by intraclass correlation coefficients (ICC) values of 24.1% and 10.5%, respectively in the optimized GLMM (Table 1). The model using a normal distribution had the lowest Akaike Information Criterion (AIC) and was further optimized by step-wise elimination of effects to minimize the AIC, resulting in the removal of target legume, length of the growing period and gender of household head as independent variables, indicating that they were not important for explaining variation in legume species richness per household.

**Table 1:** Agroecological and socioeconomic factors influencing legume species richness.

Factor	Model parameters		
**Random effects**	**variance**	**std. dev.**	**ICC**
Zone (nested in target legume)	0.14423	0.2506	0.2411
Community (nested in zone and target legume)	0.06279	0.3798	0.1049
Residual	0.39131	0.6255	0.6540
**Fixed effects**	**estimate**	**std. error**	**p-value**
Intercept	0.48010	0.29916	0.111563
Altitude (thousands of meters)	0.32787	0.12636	0.010659 *
Area planted to legumes (1/4 hectares)	0.39187	0.02133	< 2e-16 ***
Relative wealth (higher income farmers)	0.25525	0.07024	0.000292 ***
Area planted x relative wealth	-0.11621	0.02385	1.26e-06 ***

The optimized linear mixed model estimates the effects of agroecological and socioeconomic factors on the number of legume species planted per household during the 2015/16 growing season. Intraclass correlation coefficients (ICC, also known as variance partition coefficients) indicate the percent of variation in species richness explained by clusters of samples within communities and zones.

* p < 0.05

*** p < 0.001

**Fig 5:**
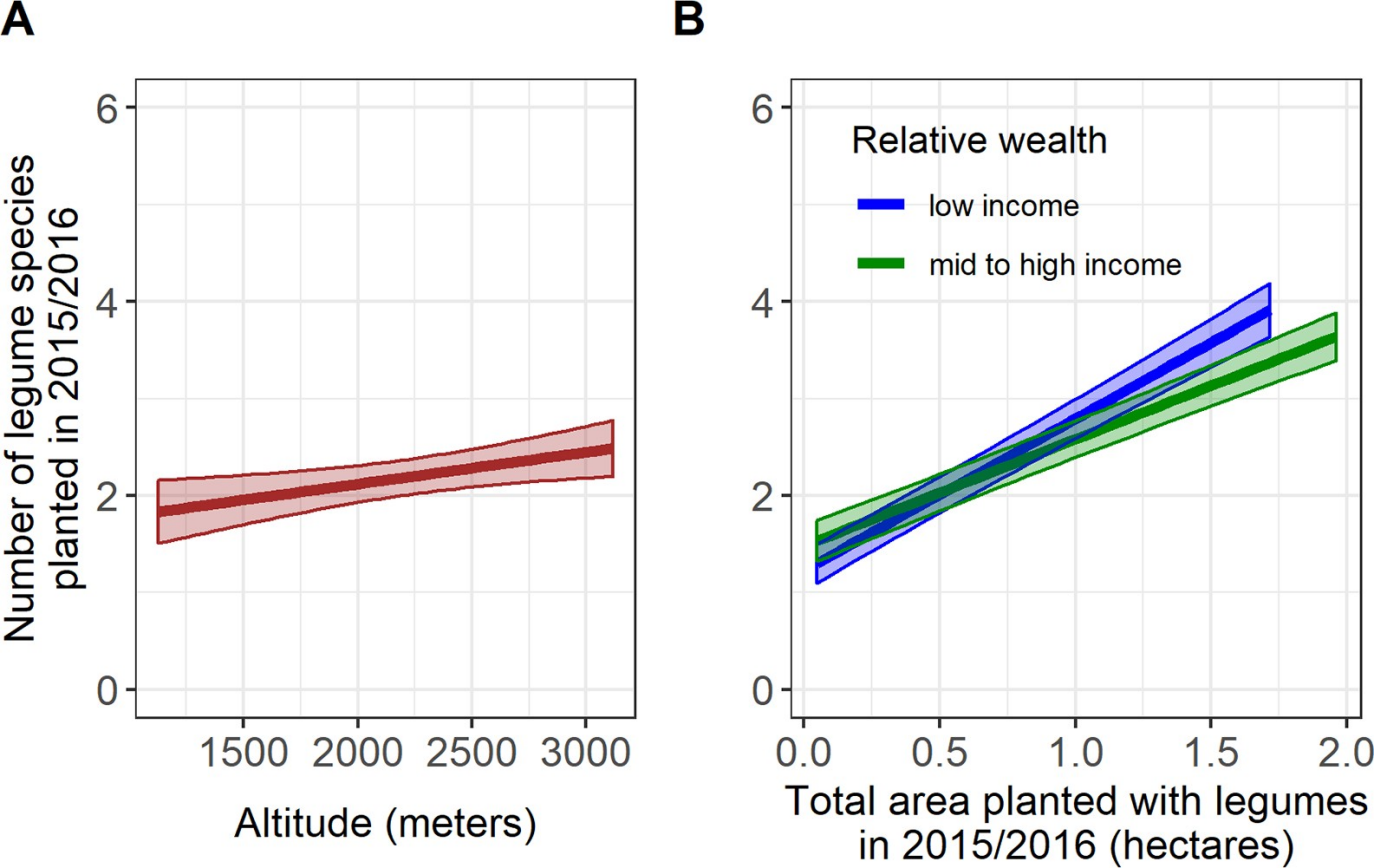
Marginal effects of factors influencing legume species richness per household. A) altitude and B) the interaction between total area planted to legumes and relative wealth; shaded areas represent 95% confidence intervals.

Altitude had a significant yet relatively small effect on species richness per household (p = 0.011), with an estimated increase of 0.33 species for every 1000-meter rise in altitude (Fig 5A). This finding is supported by the species response curves (Fig 4) that show more legume species are available at higher altitudes. A larger effect was observed for land area; farmers who devoted more land to legumes planted significantly more species (p<0.001), with an estimated increase of 1.57 species for each additional hectare. Relative wealth had a weak but significant effect on species richness (p<0.001): mid- to high-income farmers planted more legumes species than lower-income farmers in the same district and zone. Interestingly, the combination (interaction term) of relative wealth and area planted to legumes has a highly significant effect on legume species richness (p<0.001). The number of species planted increases more dramatically with land area among low-income farmers than for mid-to-high income households (Fig 5B).

### Intraspecific diversity

**Fig 6:**
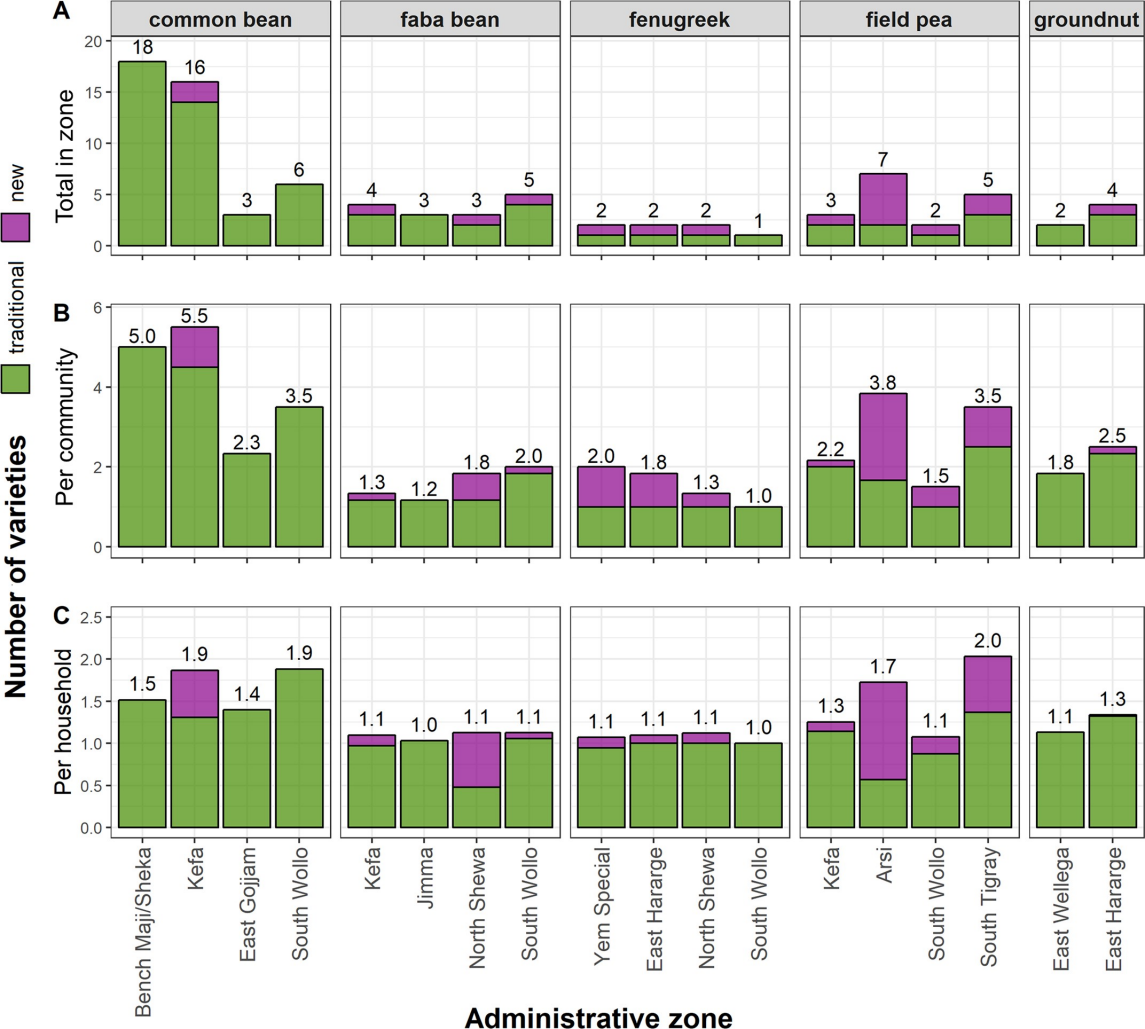
Varietal richness of legumes among households and communities in Ethiopia. A) the total number of varieties reported within the zone, B) the average number of varieties per community, and C) the average number per household in 2015/2016.

We documented 93 varieties across the five legume species (S2 Table). The number of legume varieties was highly variable according to species and zone (Fig 6A). The highest varietal richness was observed for common bean in southwestern Ethiopia, where 18 varieties were recorded in Bench Maji and Sheka and 16 in Kefa zone. In the case of Bench Maji and Sheka, all 18 varieties were identified by informants as traditional, whereas in Kefa, farmers agreed that two of their varieties were new. Across the country, in all but one zone, traditional varieties outnumbered new types; in most zones, only one new variety was ever named. The sole exception to this trend was for field pea in Arsi Zone, where farmers described five varieties as new and only two as traditional.

The average number of varieties per community ranged from 1.0 to 5.5 (Fig 6B). As at the zonal level, varietal richness per community was highest for common bean and lowest for faba bean and fenugreek. However, the average number of varieties per household was much lower (ranging from 1.0 to 2.0). Moreover, we found no correlation between the number of varieties reported within a zone or community and the number planted by farmers. For example, those farmers interviewed about common bean in Bench Maji and Sheka planted an average of 1.5 varieties, despite reports of 18 distinct varieties, whereas common bean growers in South Wollo planted an average of 2.0 varieties, even though only six were reported within the zone. As was observed at zonal and community levels, individual households were more likely to plant traditional rather than new varieties. Exceptions included faba bean growers in North Shewa and field pea growers in Arsi.

The likelihood of households planting more than one variety was highly variable according to zone and community and was rarely explained by agroecological or socioeconomic factors (Table 2). Stepwise optimization of mixed models for each species eliminated all but two of the six fixed effects in the original models. For one species (groundnut), all fixed effects were removed, and for another (faba bean) none were significant in the optimized model. Altitude, total rainfall, length of the growing season, and relative wealth were removed from all models, indicating they did not show a consistent effect on varietal richness of any of the species considered. However, the total land area planted to legumes had a large and highly-significant positive effect for common bean, fenugreek, and field pea. Gender of the household head, although included in the same three models, was only marginally significant in one of them (common bean).

**Table 2:** Varietal richness as a function of sociocultural and agroecological factors.

	Common bean	Faba bean	Fenugreek	Field pea	Groundnut
**Sample size**	263	255	275	272	83
**Random effects**	**Variance (Intraclass Correlation Coefficient)**				
Zone	0.4235 (0.0834)	0.03114 (0.00564)	13.025 (0.6697)	3.806 (0.4697)	0.8469 (0.1915)
Community	1.3640 (0.2686)	2.204 (0.3989)	3.135 (0.1612)	1.008 (0.1244)	0.2861 (0.06469)
Residual	3.290 (0.6480)	3.290 (0.5955)	3.290 (0.1691)	3.290 (0.4060)	3.290 (0.7438)
**Fixed effects**	**Estimate (p-value)**				
Intercept	-1.858 (0.00308**)	-3.799 (0.00129**	-7.293 (0.00453**	-2.247 (0.0394*)	-1.730 (0.0214*)
Area planted to legumes (ha)	1.905 (<0.001***)	0.688 (0.3095)	3.740 (0.00829**	2.029 (<0.001***)	-
Household head gender (male)	0.8481 (0.05330)	0.7804 (0.4832)	1.443 (0.1871)	-	-

Optimized General Linear Mixed Models (GLMMs) of varietal richness per household for five legume crops planted in 2015/2016.

* p < 0.05

** p < 0.01

*** p < 0.001

### Conservation status of legume varieties

**Fig 7:**
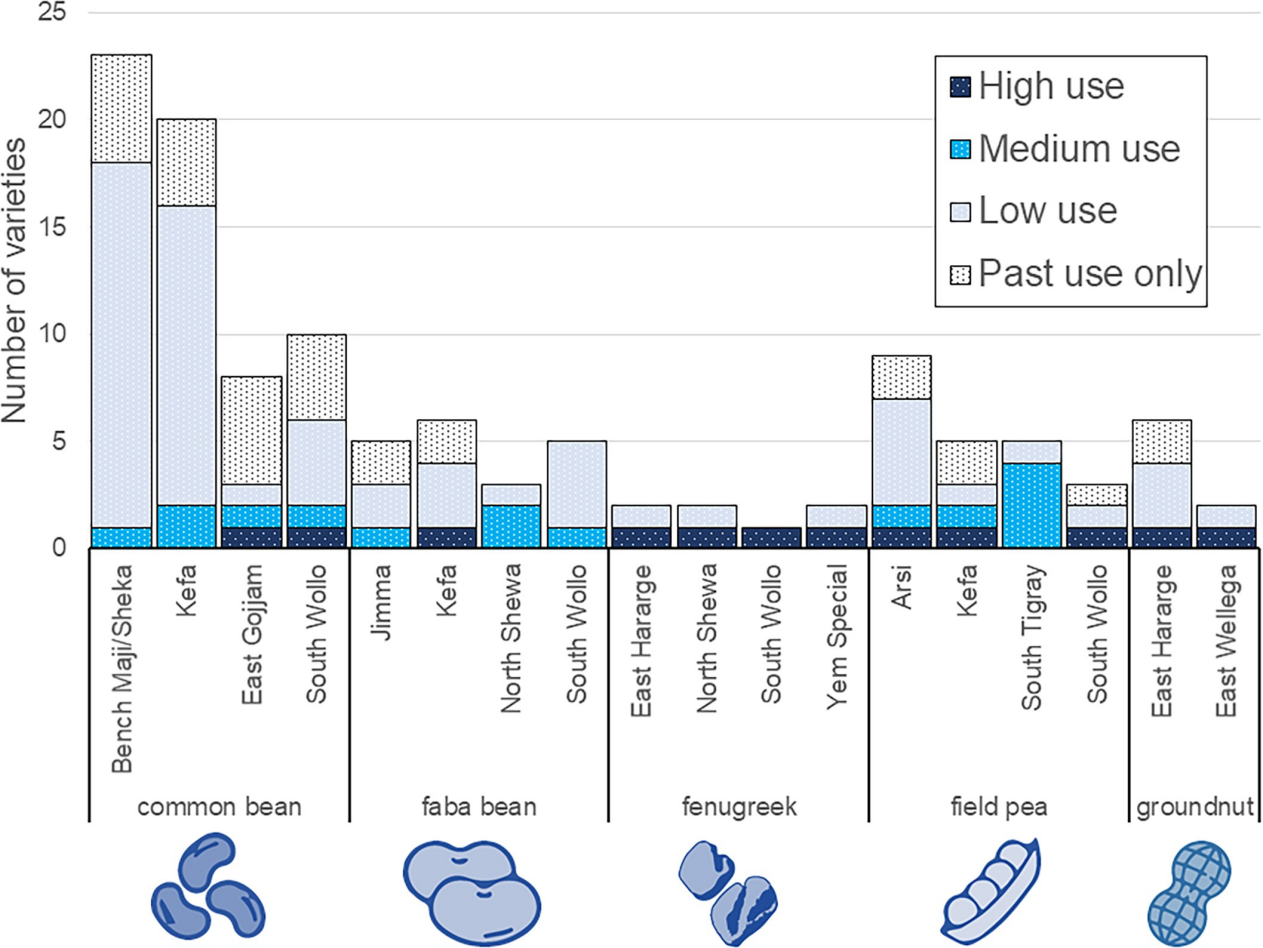
Conservation status of legume varieties. ‘Low use’ was assigned to varieties used by less than 1/3 of farmers, ‘medium use’ for those used by 1/3 to less than 2/3 of farmers, and ‘high use’ for those used by more than 2/3 of farmers in the zone during the 2015/2016 season. Varieties designated ‘past use only’ were remembered from the past, said to have disappeared, and not reported as planted by other farmers in 2015/2016.

The survey found that most legume varieties are relatively uncommon, planted by less than 1/3 of the farmers interviewed within the zone (Fig 7). Few varieties are widely planted; usually, a single variety is planted by more than 2/3 of the farmers in the zone, whereas the other varieties show medium or low use. The single exception is for groundnut in East Wellega, where two varieties showed high use. In those zones with high varietal richness, most varieties show low use. For example, of the 18 varieties of common bean reported in Bench Maji and Sheka, one was categorized as high use, two as medium use, and 15 as low use. An interesting exception was found in South Tigray, where four out of the five varieties of field pea showed medium use and the remaining one low use.

**Fig 8:**
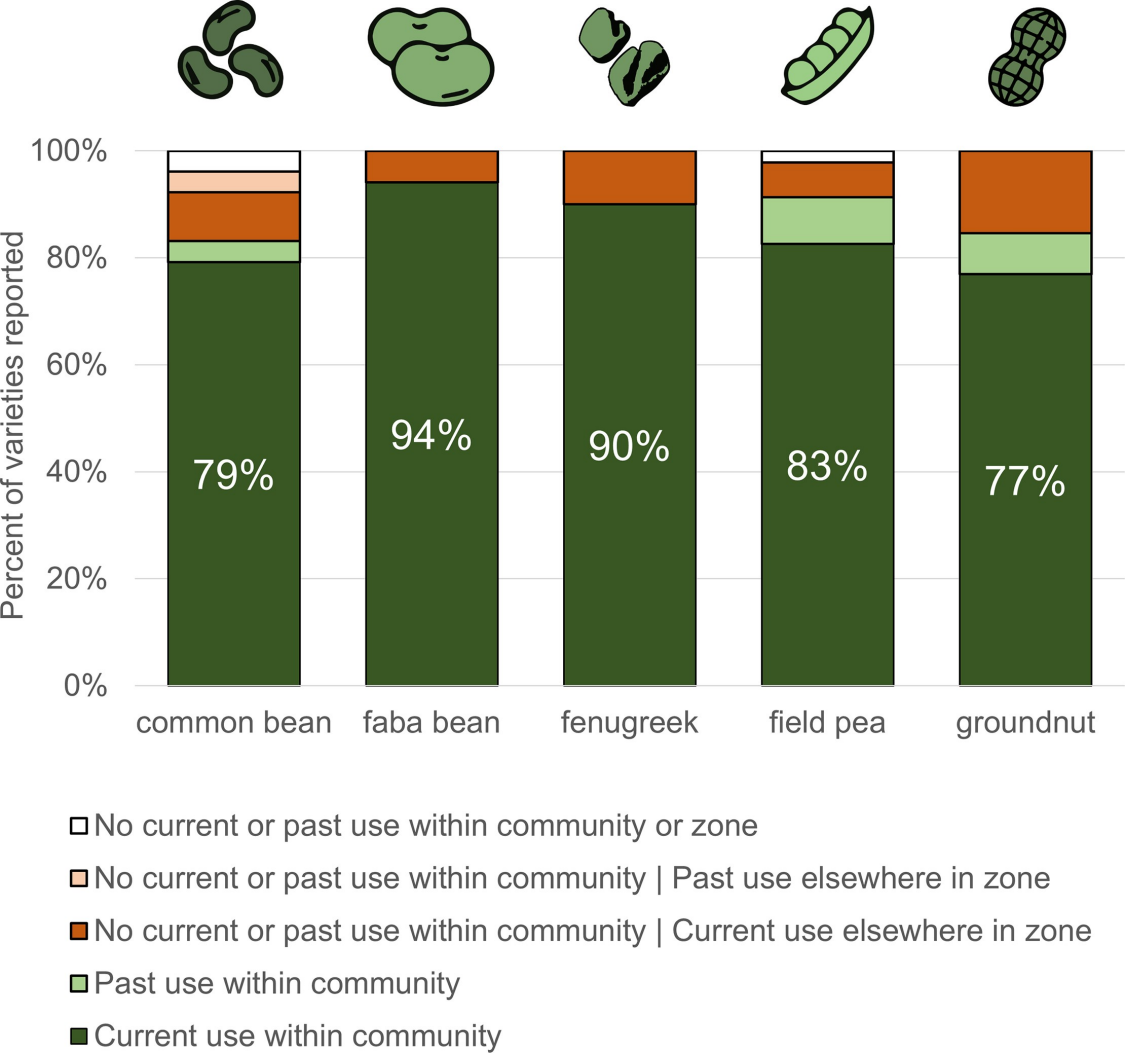
Knowledge of varieties grown by other farmers in the community. Percentage of varieties reported grown by others but not interviewees themselves. Current use refers to the 2014 to 2016 growing seasons; past use refers to any time prior to 2014.

Farmers were aware of the varieties planted by others in their community. When we asked farmers to name varieties planted by others but not themselves, we encountered those same varieties within the same community more than 77% of the time, even with a limited sample size of 12 households (Fig 8).

Farmers remembered 43 legume varieties that they claimed were no longer planted in their communities. Of these, about half (22) were reported to be planted by other farmers within the same zone. Still others (5) were said to have been planted recently, but more than three years prior to the survey. The remaining 16 varieties were never reported as planted in the same zone, although many of them were remembered by multiple farmers (Table 3). In some cases, we found varieties with the same or similar local names in other zones; but agromorphological or genetic characterization would be necessary to determine if they are indeed the same variety.

**Table 3:** Legume varieties determined to be rare or lost from zone.

Species	Administrative zone	Local name of variety	Number of informants who remembered variety
common bean	Bench Maji/Sheka	dalecha boloqe	1
	Bench Maji/Sheka	kuse	3
	East Gojjam	burabure boloqe	2
	East Gojjam	burea boloqe	1
	East Gojjam	teftafa nech boloqe	1
	East Gojjam	tikur boloqe	3
	Kefa	gote gobo	2
	Kefa	kochi gobo	3
	South Wollo	bakelo boloqe	1
	South Wollo	burea boloqe	4
faba bean	Jimma	nekelo	9
	Jimma	yeferenje baqela	3
	Kefa	shereda	7
	Kefa	welayita	2
field pea	South Wollo	gurazmen ater	1
groundnut	East Hararge	jewis	4

Varieties included in this list were reported as having disappeared from communities and were never detected in the same community or zone.

## Discussion

Farmers in Ethiopia play an important role in the conservation and management of agrobiodiversity. Our survey results confirm that a diversity of legumes is maintained by farming communities across the 107 communities included in this study. The survey documented 13 of 19 edible legume species known to be cultivated within Ethiopia. The research focused on major production areas of five species, including three highland species that were early introductions from West Asia and two lowland crops that were later introductions from the Americas [23]. Overall, the number of legume species was found to be higher at upper altitudes, as illustrated by species response curves, and supported by the linear mixed model for legume species richness per household.

The survey found the greatest varietal diversity among common bean, a lowland species first introduced by the Portuguese in the 16^th^ and 17^th^ centuries, followed by many additional introductions of new germplasm since [47]. As in other parts of Africa [48], common bean has diversified to play a wide range of roles within Ethiopian farming systems and food culture. The diversity of common bean varieties in the lowlands may substitute for lack of other legume species suitable to warmer conditions. This suggests that in maintaining on-farm diversity, farmers select legume types with distinct characteristics, regardless of whether those are different species or varieties according to Linnaean taxonomy.

Farmers frequently commented that they were unable to plant additional legume species or varieties due to the limited size of their landholdings. Our analysis also indicated that the total area planted to legumes has a significant bearing on the legume species planted per household, as well as varietal richness per household for three species (common bean, fenugreek and field pea). On the one hand, mid-to-high income households tend to devote more land to legumes–presumably, because they have access to more land–and are therefore more likely to plant a greater number of legume species and/or varieties. On the other hand, when land planted to legumes is equal, low income farmers plant more species than those with mid-to-high income. There are several plausible explanations for this finding: i) Low-income farmers may be more likely to use diversity to mitigate the risk of crop failure. ii) Low-income farmers are more likely to grow most of their own food, therefore on-farm diversity is necessary to meet a range of dietary needs. Legume diversity is particularly important to farmers who cannot afford meat as their primary source of protein. By contrast, farmers with mid- to high-incomes are perhaps more likely to purchase food from the market to meet their dietary needs. iii) Low income farmers are less able to afford chemical fertilizers, and so legumes are essential sources of soil fertility. Given that landholdings tend to be fragmented across heterogeneous landscapes [49], farmers who rely on legumes will use different species or varieties in each of their fields. For example, plots in irrigated bottomlands are more likely to experience frost and flooding, whereas hilltops are more likely to face lower temperatures and hail, requiring different crops to fit each condition.

Ethiopian farmers appear to favor traditional over newly introduced varieties. Across all species and almost every zone, the number of traditional varieties far exceeds that of new types. Although the survey did not determine whether farmers are replacing traditional varieties with newer ones, some are planting a combination of both. Rarely are new varieties dominant; the only exceptions are for field pea in Arsi and faba bean in North Shewa, both areas known for mechanization of their farming systems and rapid adoption of introduced varieties developed at nearby research facilities. An important area for further investigation would be the traits and values associated with each crop–including traditional and new varieties–and which of those influence their frequency of use within farming communities.

One of the primary advantages of traditional varieties (of which most are landraces) is that they are well-adapted to specific agroecological conditions [50,51]. The heterogeneity of Ethiopian landscapes results in most varieties being planted by a small number of farmers working in similar microclimatic and edaphic conditions. Given the rarity of most varieties, community-level coordination is required to avoid inadvertent losses. Our analyses indicate that farmers know about the varieties planted by other farmers within their community, including those they do not plant themselves. Almost always, when a farmer named a variety planted by others, we were able to confirm that it was currently or recently planted in the same community. Such knowledge is an important component of farmers’ adaptive capacity, as they know where to find alternatives whenever conditions change. Moreover, awareness of the varieties within their community allows families to monitor their use and ensure that they remain available. Hence, the maintenance of legume diversity within communities is a collective endeavor.

In addition to the collective efforts of farmers, formal institutions play major roles in the conservation of Ethiopia’s legume diversity. *Ex situ* conservation by the national gene bank of the EBI can ensure the survival of germplasm, but the use of new varieties by future farmers will require sharing knowledge about seeds. One of EBI’s primary aims is to broaden the availability of genetic resources through collection and distribution of landraces; at the same time, EBI can work with farmers to document their knowledge about morphological, agronomic and culinary traits.

EBI and civil society organizations (e.g., Ethio-Organic Seed Action) have established community-based seed banks and libraries from which farmers are able to ‘borrow’ seeds if they contribute some of the varieties they are planting [52]. These institutions represent a promising way to maintain legume diversity within communities, while at the same time involving farmers in the collection of landraces to be stored as germplasm resources. Seed banks may be particularly important in the restoration of agrobiodiversity. For example, we have identified varieties that have disappeared from communities; the local names in Table 3 were provided to the EBI to see if existing collections might include them. Those that are found can be returned to the communities that have lost them.

Finally, policy-makers need to consider the inadvertent impacts of agricultural development strategies on legume diversity. For example, in promoting the use of chemical fertilizers, extension systems may unintentionally discourage farmers from using legumes in crop rotation, with undesirable impacts on household nutrition and soil quality. We advocate a balanced approach by which extension agents and farmers discuss the advantages of fertilizer use alongside the short- and long-term benefits of legume diversity. Furthermore, extension agents are uniquely positioned to monitor legume diversity and to work with farmers in their communities to ensure varieties are available to households interested in cultivating them. One promising initiative is Legume CHOICE, a project supported by the International Livestock Research Institute aimed at identifying legume options based on farmers’ needs, assets, and agroecological context [53]. The inclusion of a wider array of traditional varieties in such a program could expand farmers’ abilities to adapt to new and more variable conditions.

## Conclusions

Through a coordinated investigation of legume diversity across many of Ethiopia’s diverse farming communities, we found that species richness of legumes was unevenly distributed among administrative zones and agroecologies. Legume species richness per farming household was generally greater at higher altitudes and among farmers who planted more land to legumes; lower-income farmers tend to plant more species on the same area of land. The number of varieties detected per zone was highly variable, but traditional varieties were more common. Even in cases where more than 10 varieties were found within a zone, the number of varieties per household was consistently low, averaging between one and two. The only factor found to have a significant effect on varietal richness was area devoted to legumes. Most varieties are planted by a small fraction of farmers, and 16 varieties were reported to have disappeared. However, farmers know about the varietal diversity within their communities, and are able to name those planted by their neighbors.

These results indicate that individual households conserve only a small part of legume diversity, and it is through collective awareness and action that species and varieties are maintained. Looking forward, it is important to strengthen community structures that monitor and maintain legumes for farming families so that they have options when conditions change. Strategic investments in traditional seed exchange networks, community seed banks, showcase trials, and rigorous documentation of varietal traits can enhance the conservation of legumes for future generations of farmers and improve their capacity to adapt to change.

## Supporting information

S1 Appendix Structured interview protocol using Open Data Kit (ODK). Interviews were conducted using the ODK Collect application on a GPS-enabled mobile phone. All dates are written according to the Ethiopian Calendar.
(DOCX)

S1 Data Structured interview data. Includes species richness, varietal richness, agroecological and socioeconomic data associated with farming households interviewed. Names and other identifying information have been removed to ensure the confidentiality of human subjects.
(XLSX)

S2 Data Status of varieties. Based on analysis of structured survey data: number of farmers reporting current, recent, and past use of each variety, as well as number of farmers reporting use of a variety by others in their community.
(XLSX)

S1 Table Summary of structured interviews according to administrative zones and agroecologies. (DOCX)

S2 Table Varieties of legumes. The varieties of legume crop species documented according to region and administrative zone, including local vernacular name, percent of general informants who reported planting between 2014 and 2016 (n = 72 per zone), and identification as a traditional or new variety.
(DOCX)
