# Supplementary material for: Inter- and intraspecific diversity of food legumes among households and communities in Ethiopia
Source: PLoS One. 2019 Dec 23;14(12):e0227074. doi: 10.1371/journal.pone.0227074 (PMC6927635; doi:10.1371/journal.pone.0227074)
Supplement: S1 Appendix — Interviews were conducted using the ODK Collect application on a GPS-enabled mobile phone. All dates are written according to the Ethiopian Calendar. (DOCX) [file pone.0227074.s001.docx]

# S1 Appendix. Structured interview protocol using Open Data Kit (ODK). Interviews were conducted using the ODK Collect application on a GPS-enabled mobile phone. All dates are written according to the Ethiopian Calendar.

## Section 1: Basic information *(Italicized items will be generated automatically in ODK)*

| *Date of interview:* | *Start time of interview:* |
| --- | --- |
| Name of interviewer: | |
| Region: | Zone: |
| Wereda/District: | Kebele/Sub-District: |
| *Latitude (in decimal degrees):* | *Longitude (in decimal degrees):* |
| *Altitude (in meters):* | |
| Agroecological classification (e.g. H1, SH2): | |
| Random number of household from farmers’ association list: | |
| Relative wealth on farmers’ association list: ☐ Low income ☐ Middle/High income | |

## Section 2: General information about informant

| Name of informant: |
| --- |
| Language spoken by informant during interview: ☐ Amharic ☐ Other (specify): __________ |
| Age (**observed**): ☐ 18 to 30 ☐ 30 to 45 ☐ 45 to 60 ☐ 60+ |
| Gender (**observed**): ☐ Male ☐ Female |
| Is the informant the household head? ☐ Yes ☐ No |
| 🡪If no, household-head gender: ☐ Female-headed ☐ Male-headed |

## Section 3: Market Access

| How far is the **closest** market from your home? ___ minutes on foot |
| --- |
| Do you go to any other **larger** market? ☐ Yes ☐ No |
| 🡪If yes, how far is this **larger** market from your home? ___ minutes (on foot) |
| How far is the nearest **road with vehicle transport** from your home? ___ minutes (on foot) |

## Section 4: Interspecific diversity of legume crops

| Are you growing any legumes this year? ☐ Yes ☐ No *If yes, select on list and ask about hectares.* |
| --- |
| Which legumes did you **plant** in 2008 (Meskerem to Pagume)? |
| For each of the legumes you listed, how many hectares of land did you **plant** in 2008 (Meskerem to Pagume) including any areas under intercropping? *Make sure you convert any local units to hectares* (e.g. 1 timad or qert = 0.25 hectare). |

## Section 5: Use of [legume of interest]

| How do you use [legume of interest]? ☐ Food ☐ Spice ☐ Medicine ☐ Fodder/Forage ☐ Fuel  ☐ Market ☐ Bee forage ☐ Other (specify): ________ |
| --- |

## Section 6: Cropping Practices

| Do you rotate [legume of interest] with other crops? ☐ Yes ☐ No |
| --- |
| 🡪If yes, with which crops do you rotate with [legume of interest]? |
| 🡪 If yes, how often do you plant [legume of interest] within the crop sequence? |
| Do you intercrop [legume of interest] with other crops? ☐ Yes ☐ No |
| 🡪If yes, with which crops do you plant [legume of interest] in the same field? |

## Section 7: Intraspecific diversity of [legume of interest]

**Note:** Be sure to include all varieties, including landraces and released/improved types.

| What varieties of [legume of interest] have you grown in the past three years (for the 2007, 2008, and 2009 harvests)? |
| --- |
| Are there any other varieties that you have grown in the past, prior to 2007? |
| Are there any other varieties of [legume of interest] grown by other farmers in your community, but not by yourself? |
| Are there any other varieties of [legume of interest] that you remember from a long time ago, or have heard about from Elders, that are no longer grown by your community? |
| If you run out of a particular variety, whom would you ask to replenish your seeds? (*Record name as a potential key informant*). |

## Section 8: Key attributes of varieties

**Instructions:** At the top of each column, write the names of all varieties harvested in 2007, 2008, and 2009. Use additional sheets if necessary

| Question | Variety 1 | Variety 2 | Variety 3 | Variety 4 |
| --- | --- | --- | --- | --- |
| Local vernacular name of variety (copied) |  |  |  |  |
| Is this a **traditional** or a **new** variety? |  |  |  |  |
| **Where** did you first obtain this variety? (e.g. family, neighbors*, DA, market, food aid, research center) |  |  |  |  |
| For **how many years** have you planted this variety? |  |  |  |  |
| **Where** do you plant this variety? (e.g. main fields, field margins, home gardens, fence lines, other places) |  |  |  |  |
| Are the places where you grow this variety **rainfed, irrigated, or both**? |  |  |  |  |
| Do you plant this variety on **soils with low, moderate, and/or high fertility**? |  |  |  |  |
| How many **times per year** do you sow this variety? |  |  |  |  |
| When do you usually **sow** this variety? |  |  |  |  |
| When do you usually **harvest** this variety? |  |  |  |  |
| Is this variety grown mainly for **home use** or mainly for the **market**? |  |  |  |  |
| How many hectares of these varieties did you harvest in 2008 and 2007, including area under intercropping? *Make sure you convert any local units to hectares* (e.g. 1 timad or qert = 0.25 hectare). | 2008:  2007: | 2008:  2007: | 2008:  2007: | 2008:  2007: |
| How much of this variety did you harvest in 2008 and 2007? *Record with local units to be converted later*. *Be sure to include the name of the units. Later, go to a local market to determine the conversion factor from local units to kilograms.* | 2008:  2007: | 2008:  2007: | 2008:  2007: | 2008:  2007: |
| Based on your experience, was the yield for this variety in 2008 and 2007 a very high yield, a high yield, a medium yield, a low yield, or a very low yield? | 2008:  2007: | 2008:  2007: | 2008:  2007: | 2008:  2007: |
| What is the current **price** of this variety at your local market? (use farmer units) |  |  |  |  |

* If a particular variety came from friends or family, ask for the name of the individual who provided it as a potential key informant. Record that person’s name in your notebook.

## Section 9: Rating of attributes for varieties of [legume of interest]. Ask the informant to rate the varieties of [legume of interest] planted for the 2007, 2008, and 2009 harvests. This is NOT a ranking activity, so multiple varieties may receive the same score.

| Rating criteria and scale | Var. 1 | Var. 2 | Var. 3 | Var. 4 | Var. 5 |
| --- | --- | --- | --- | --- | --- |
| **Local name** (copied from previous pages) |  |  |  |  |  |
| **Best yield this variety has ever given you** (5=very high, 4=high, 3=medium, 2=low, 1=very low) |  |  |  |  |  |
| **Yield under drought conditions** (5=very high, 4=high, 3=average, 2=low, 1=very low) |  |  |  |  |  |
| **Yield when excessive rain causing water logging** (5=very high, 4=high, 3=average, 2=low, 1=very low) |  |  |  |  |  |
| **Yield when the growing season is short (due to late start or early finish of rainy season)** (5=very high, 4=high, 3=average, 2=low, 1=very low) |  |  |  |  |  |
| **Resistance to frost** (5=never affected by frost, 4=rarely affected by frost, 3=sometimes affected by frost, 2=often affected by frost, 1=always affected by frost) |  |  |  |  |  |
| **Resistance to common diseases** (5=never affected by diseases, 4=rarely affected, 3=sometimes affected, 2=often affected, 1=always affected by diseases) |  |  |  |  |  |
| **Tolerance to common insect pests** (5=never affected by insect pests, 4=rarely affected, 3=sometimes affected, 2=often affected, 1=always affected) |  |  |  |  |  |
| **Tendency for seeds to detach/shatter** (5=seeds never detach/shatter 4=rarely detach/shatter, 3=detach/shatter about half the time, 2=often detach/shatter, 1=always detach/shatter) |  |  |  |  |  |
| **Importance as food for the household** (5=extremely important, 4=very important, 3=somewhat important, 2=not so important, 1=not at all important) |  |  |  |  |  |
| **Importance as a source of income** (5=extremely important, 4=very important, 3=somewhat important, 2=not so important, 1=not at all important) |  |  |  |  |  |
| **Importance as fodder for livestock** (5=extremely important, 4=very important, 3=somewhat important, 2=not so important, 1=not at all important) |  |  |  |  |  |
| **Effect on soil fertility** (5=very positive effect on soil fertility, 4=some positive effect, 3=no effect, 2=some negative effect, 1=very negative effect on soil fertility) |  |  |  |  |  |
| **Ease of harvesting** (5=Very easy to harvest, 4=easy, 3=neither easy nor difficult, 2=somewhat difficult, 1=very difficult to harvest) |  |  |  |  |  |
| **Ease of preparing as food** (5=very easy to harvest, 4=easy, 3=neither easy nor difficult, 2=somewhat difficult, 1=very difficult to harvest) |  |  |  |  |  |
| **Taste** (5=very good taste, 4=good taste, 3=neither good nor bad taste, 2=bad taste, 1=very bad taste) |  |  |  |  |  |

## Section 10: Gender roles in production and management of [legume of interest]

| Which gender and age groups live in your household (including the respondent)? | ☐ M-children  ☐ F-children | ☐ M-adults  ☐ F-adults | ☐ M-Elders  ☐ F-Elders | ☐ Yes  ☐ No |
| --- | --- | --- | --- | --- |

When it comes to [legume of interest], who within the family usually participates in the following activities? Check any that apply.

| Activity | Gender and age groups* | | | Is this true for all varieties?* |
| --- | --- | --- | --- | --- |
| Ploughing with animals (mares) | ☐ M-children  ☐ F-children | ☐ M-adults  ☐ F-adults | ☐ M-Elders  ☐ F-Elders | ☐ Yes  ☐ No |
| Preparing the soil by hand with a hoe (mekofer) | ☐ M-children  ☐ F-children | ☐ M-adults  ☐ F-adults | ☐ M-Elders  ☐ F-Elders | ☐ Yes  ☐ No |
| Leveling soil and removing uprooted weeds (gulgualo) | ☐ M-children  ☐ F-children | ☐ M-adults  ☐ F-adults | ☐ M-Elders  ☐ F-Elders | ☐ Yes  ☐ No |
| Sowing (zer mezerat) | ☐ M-children  ☐ F-children | ☐ M-adults  ☐ F-adults | ☐ M-Elders  ☐ F-Elders | ☐ Yes  ☐ No |
| Weeding (marem) and cultivation (kutkuato) | ☐ M-children  ☐ F-children | ☐ M-adults  ☐ F-adults | ☐ M-Elders  ☐ F-Elders | ☐ Yes  ☐ No |
| Fertilizer application (madaberia, fig, kompost…) | ☐ M-children  ☐ F-children | ☐ M-adults  ☐ F-adults | ☐ M-Elders  ☐ F-Elders | ☐ Yes  ☐ No |
| Harvesting (mached ena mesebseb) | ☐ M-children  ☐ F-children | ☐ M-adults  ☐ F-adults | ☐ M-Elders  ☐ F-Elders | ☐ Yes  ☐ No |
| Threshing (mewkat) | ☐ M-children  ☐ F-children | ☐ M-adults  ☐ F-adults | ☐ M-Elders  ☐ F-Elders | ☐ Yes  ☐ No |
| Storage (makemachet) | ☐ M-children  ☐ F-children | ☐ M-adults  ☐ F-adults | ☐ M-Elders  ☐ F-Elders | ☐ Yes  ☐ No |
| Marketing (meshet) | ☐ M-children  ☐ F-children | ☐ M-adults  ☐ F-adults | ☐ M-Elders  ☐ F-Elders | ☐ Yes  ☐ No |
| Seed selection (zer memret) | ☐ M-children  ☐ F-children | ☐ M-adults  ☐ F-adults | ☐ M-Elders  ☐ F-Elders | ☐ Yes  ☐ No |
| Food preparation (megib mazegajet) | ☐ M-children  ☐ F-children | ☐ M-adults  ☐ F-adults | ☐ M-Elders  ☐ F-Elders | ☐ Yes  ☐ No |
| Collection of fodder for animals (meno mesebsebe) | ☐ M-children  ☐ F-children | ☐ M-adults  ☐ F-adults | ☐ M-Elders  ☐ F-Elders | ☐ Yes  ☐ No |
| Other activity (specify): | ☐ M-children  ☐ F-children | ☐ M-adults  ☐ F-adults | ☐ M-Elders  ☐ F-Elders | ☐ Yes  ☐ No |

* Note: For this classification only, **children** are individuals 14 and younger, **adults** are ages 15 to 59, and **Elders** are 60 or older.

** Take detailed notes of any exceptions for particular varieties

## Section 11: Closing and follow-up questions

| *Ask if you haven’t recorded any names of potential key informants in your notebook:* We are looking for both men and women who have a lot of knowledge about different varieties of [legume of interest]. Is there anyone from your community who you recommend? | |
| --- | --- |
| Thank you very much for answering my questions. Do you have any comments and/or questions **you would like to raise** at this time? (Record farmers’ questions in your notebook, if relevant.) | |
| At any point during the interview, did the informant indicate that s/he had gained some new knowledge? ☐ Yes ☐ No | |
| 🡪 If yes, what knowledge did he/she report to have gained as a result of the interview (Record farmers’ statements in your notebook.) | |
| *Time interview was completed:* | *Duration of interview (minutes):* |

Additional notes:
