# Supplementary material for: Inter- and intraspecific diversity of food legumes among households and communities in Ethiopia
Source: PLoS One. 2019 Dec 23;14(12):e0227074. doi: 10.1371/journal.pone.0227074 (PMC6927635; doi:10.1371/journal.pone.0227074)
Supplement: S1 Table — (DOCX) [file pone.0227074.s004.docx]

**S1 Table. Summary of structured surveys according to agroecological and administrative zones.**

| **Temperature and altitude** | **Humidity (length of growing period)** | **AEZ Code** | **Administrative zone (Region)** | **Legume species**  **(# informants)** |
| --- | --- | --- | --- | --- |
| warm lowlands  (500 to 1600 masl) | sub-moist (61 to 120 days) | SM2 | East Hararge (Oromiya) | groundnut (36) |
|  | moist (121-180 days) | M2 | East Gojjam (Amhara) | common bean (36) |
|  |  |  | South Wollo (Amhara) | common bean (36) |
|  |  |  | East Hararge (Oromiya) | groundnut (36) |
|  |  |  | East Wellega (Oromiya) | groundnut (36) |
|  | sub-humid (181 to 240 days) | SH2 | East Hararge (Oromiya) | fenugreek (36) |
|  |  |  | East Wellega (Oromiya) | groundnut (36) |
|  |  |  | Yem Special (SNNP) | fenugreek (36) |
|  | per-humid (more than 300 days) | PH2 | Bench Maji & Sheka (SNNP) | common bean (36) |
| tepid mid-highlands (1600 to 2400 masl) | sub-moist  (61 to 120 days) | SM3 | North Shewa (Amhara) | faba bean (36) |
|  |  |  |  | fenugreek (36) |
|  |  |  | South Wollo (Amhara) | common bean (36) |
|  |  |  |  | faba bean (36) |
|  |  |  |  | fenugreek (36) |
|  |  |  |  | field pea (36) |
|  |  |  | South Tigray (Tigray) | field pea (36) |
|  | moist (121-180 days) | M3 | East Gojjam (Amhara) | common bean (36) |
|  |  |  | Arsi (Oromiya) | field pea (36) |
|  | sub-humid (181 to 240 days) | SH3 | East Hararge (Oromiya) | fenugreek (36) |
|  |  |  | Jimma (Oromiya) | faba bean (36) |
|  |  |  | Kefa (SNNP) | field pea (36) |
|  |  |  | Yem Special (SNNP) | fenugreek (36) |
|  | humid (241 to 300 days) | H3 | Arsi (Oromiya) | field pea (36) |
|  |  |  | Jimma (Oromiya) | faba bean (36) |
|  |  |  | Kefa (SNNP) | common bean (36) |
|  |  |  |  | faba bean (36) |
|  |  |  |  | field pea (36) |
|  |  |  | Bench Maji & Sheka (SNNP) | common bean (36) |
|  | per-humid (more than 300 days) | PH3 | Kefa (SNNP) | common bean (36) |
|  |  |  |  | faba bean (36) |
| cool mid-highlands (2400 to 3200 masl) | sub-moist  (61 to 120 days) | SM4 | North Shewa (Amhara) | faba bean (36) |
|  |  |  |  | fenugreek (36) |
|  |  |  | South Wollo (Amhara) | faba bean (36) |
|  |  |  |  | fenugreek (36) |
|  |  |  |  | field pea (36) |
|  |  |  | South Tigray (Tigray) | field pea (36) |
