# Supplementary material for: Inter- and intraspecific diversity of food legumes among households and communities in Ethiopia
Source: PLoS One. 2019 Dec 23;14(12):e0227074. doi: 10.1371/journal.pone.0227074 (PMC6927635; doi:10.1371/journal.pone.0227074)
Supplement: S2 Table — The varieties of legume crop species documented according to region and administrative zone, including local vernacular name, percent of general informants who reported planting between 2014 and 2016 (n = 72 per zone), and identification as a traditional or new variety. (DOCX) [file pone.0227074.s005.docx]

**S2 Table. Varieties of legumes**. The varieties of legume crop species documented according to region and administrative zone, including local vernacular name, percent of general informants who reported planting between 2014 and 2016 (n=72 per zone), and identification as a traditional or new variety.

| Species | Region | Zone | Local name of variety | Farmers planting (%) | Traditional or new (% who agree) |
| --- | --- | --- | --- | --- | --- |
| common bean | Amhara | East Gojjam | Nech Boloqe | 96% | trad. (68%) |
|  |  |  | Key Boloqe | 43% | trad. (65%) |
|  |  |  | Dalecha Boloqe | 6% | trad. (100%) |
|  |  | South Wollo | Nech Boloqe | 96% | trad. (91%) |
|  |  |  | Key Boloqe | 56% | trad. (90%) |
|  |  |  | Dalecha Boloqe | 28% | trad. (65%) |
|  |  |  | Tikur Boloqe | 17% | trad. (100%) |
|  |  |  | Gureza Boloqe | 1% | trad. (100%) |
|  |  |  | Yareba Ken Boloqe | 1% | trad. (100%) |
|  | SNNP | Bench Maji / Sheka | Chele Gobo | 53% | trad. (100%) |
|  |  |  | Ao Gobo | 25% | trad. (100%) |
|  |  |  | Nacho Gobo | 13% | trad. (100%) |
|  |  |  | Burabure Boloqe | 6% | trad. (100%) |
|  |  |  | Folfole | 6% | trad. (100%) |
|  |  |  | Bicho Defo | 4% | trad. (100%) |
|  |  |  | Bori Dori | 4% | trad. (100%) |
|  |  |  | Ye'Enchat Boloqe | 4% | trad. (100%) |
|  |  |  | Dalicha Folfole | 3% | trad. (100%) |
|  |  |  | Dalicha Koto Defe | 3% | trad. (100%) |
|  |  |  | Kanji Defo | 3% | trad. (100%) |
|  |  |  | Sologe | 3% | trad. (100%) |
|  |  |  | Tenkre | 3% | trad. (100%) |
|  |  |  | Dalicha Ao Folfole | 1% | trad. (100%) |
|  |  |  | Dingeri | 1% | trad. (100%) |
|  |  |  | Gobuap | 1% | trad. (100%) |
|  |  |  | Pantarkn | 1% | trad. (100%) |
|  |  |  | Polpole | 1% | trad. (100%) |
|  |  | Kefa | Chele Gobo | 49% | trad. (94%) |
|  |  |  | Nasir | 33% | new (100%) |
|  |  |  | Mchimiyate | 31% | trad. (100%) |
|  |  |  | Awash 01 | 19% | new (100%) |
|  |  |  | Ao Gobo | 17% | trad. (100%) |
|  |  |  | Chele Yure | 4% | trad. (100%) |
|  |  |  | Key Bure Mchimiyate | 4% | trad. (100%) |
|  |  |  | Dalicha Key Mchimyate | 3% | trad. (100%) |
|  |  |  | Manache Gobo | 3% | trad. (100%) |
|  |  |  | Shashemene | 3% | trad. (100%) |
|  |  |  | Tikur Bure | 3% | trad. (100%) |
|  |  |  | Wele Gobo | 3% | trad. (100%) |
|  |  |  | Dalicha Bure Mchimiyate | 1% | trad. (100%) |
|  |  |  | Dalicha Key Bure Mchimiyate | 1% | trad. (100%) |
|  |  |  | Dalicha Nech Mchimiyate | 1% | trad. (100%) |
|  |  |  | Nache Yure | 1% | trad. (100%) |
| faba bean | Amhara | North Shewa | Yeweha Baqela | 61% | new (68%) |
|  |  |  | Yehabesha Baqela | 40% | trad. (100%) |
|  |  |  | Yeferenje Baqela | 15% | trad. (100%) |
|  |  |  | mixture | 1% | trad. (100%) |
|  |  | South Wollo | Yehabesha Baqela | 58% | trad. (100%) |
|  |  |  | Bungn | 25% | trad. (94%) |
|  |  |  | Yeferenje Baqela | 18% | trad. (100%) |
|  |  |  | Mert | 7% | new (100%) |
|  |  |  | Enate | 4% | trad. (100%) |
|  | Oromiya | Jimma | Baqela | 63% | trad. (100%) |
|  |  |  | Arebe | 28% | trad. (70%) |
|  |  |  | Shone (mixture) | 10% | trad. (86%) |
|  |  |  | Orome | 4% | trad. (100%) |
|  | SNNP | Kefa | Ato | 72% | trad. (100%) |
|  |  |  | Dagaga | 17% | trad. (100%) |
|  |  |  | Cambata | 13% | new (100%) |
|  |  |  | Tenya | 8% | trad. (83%) |
| fenugreek | Amhara | North Shewa | Abish (Tikur Abish) | 99% | trad. (99%) |
|  |  |  | Nech Abish | 18% | new (85%) |
|  |  | South Wollo | Abish | 97% | trad. (100%) |
|  | Oromiya | East Hararge | Hulbata Guracha | 99% | trad. (97%) |
|  |  |  | Hulbata Dima | 10% | new (71%) |
|  | SNNP | Yem Special | Abesha Abish | 94% | trad. (99%) |
|  |  |  | Orome Abesh | 13% | new (100%) |
| field pea | Amhara | South Wollo | Dalicha Ater | 89% | trad. (100%) |
|  |  |  | Nech Ater | 21% | new (100%) |
|  | Oromiya | Arsi | Akuri | 71% | new (100%) |
|  |  |  | Dangalo | 43% | trad. (100%) |
|  |  |  | Tegegnech | 29% | new (100%) |
|  |  |  | Adii | 14% | trad. (100%) |
|  |  |  | Birkitu | 8% | new (100%) |
|  |  |  | Markos | 6% | new (100%) |
|  |  |  | Bilalo | 1% | new (100%) |
|  | SNNP | Kefa | Bono | 74% | trad. (100%) |
|  |  |  | Aa'O | 39% | trad. (100%) |
|  |  |  | Akuri | 11% | new (100%) |
|  |  |  | Sargagna (mixture) | 7% | trad. (100%) |
|  | Tigray | South Tigray | Tegegnech | 61% | new (100%) |
|  |  |  | Gotate Adi | 53% | trad. (100%) |
|  |  |  | Dekoko | 47% | trad. (100%) |
|  |  |  | Dekik Gotate | 38% | trad. (63%) |
|  |  |  | Hiwshilshal (mixture) | 14% | new (80%) |
|  |  |  | Birkitu | 1% | new (100%) |
| groundnut | Oromiya | East Hararge | Oldhale (Ejja) | 89% | trad. (100%) |
|  |  |  | Sartu (Sara) | 31% | trad. (100%) |
|  |  |  | Mixture | 10% | trad. (100%) |
|  |  |  | Jambo (Yambo) | 3% | trad. (100%) |
|  |  |  | Roba | 1% | new (100%) |
|  |  | East Wellega | Bure | 100% | trad. (100%) |
|  |  |  | Dima (Qay) | 15% | trad. (100%) |
